# Supplementary material for: Patterns of Intron Gain and Loss in Fungi
Source: PLoS Biol. 2004 Nov 30;2(12):e422. doi: 10.1371/journal.pbio.0020422 (PMC532390; doi:10.1371/journal.pbio.0020422)
Supplement: Table S1 — Also available at http://genes.mit.edu/NielsenEtAl/. (4.3 MB ZIP). [file pbio.0020422.st001.zip › NielsenEtAl/html/1378.html]

AN2493.1.NCU01376.1.MG04900.1.FG07375.1


```
 CLUSTAL W (1.82) Multiple Sequence Alignments - Introns Inserted


Sequence 1: NCU01376.1	668 aa
Sequence 2: MG04900.1	668 aa
Sequence 3: FG07375.1	654 aa
Sequence 4: AN2493.1	663 aa
Alignment Length: 677 aa
Number Identitical Residues: 322 aa
Alignment Score (without introns) 16198


MG04900.1 	MVFRTSFALAAAAAFLLPSTQGQTFQRLGSCPDLGCILPPDQ~SD0FLPGQFFDIRLEIH
NCU01376.1	MISSRISGALALLATSLSVVSGQTFQRLGTCPTLGCILPPDQ~QD~FLPGQHFDIRFEVH
FG07375.1 	---MVKFNTIAAFAASIAAASAQTYQRLGTCPTLGCVLPPDQ~SD~FLPGQLFDFRVEVH
AN2493.1  	----MKPTSFAGVALFASGAFGQYFQRLGGCPSLGCVFPPDQ2AD~FLPGQHFDIRLEVH
          	          *  *   . . .* :**** ** ***::****  * ***** **:*.*:*

MG04900.1 	APVNGSEAFNNGVPDEDFTVTITKDGSGAESKTITQFFSKEEPKIEKWTFNWFED~LFAE
NCU01376.1	APKNGSEAFNDGVPDEKFTVTIAKDN--GKPQSIASFFELKEPSLEKWTFSWYED~LFAQ
FG07375.1 	APVNGSEAAHDGKPDQKFKVTIAKEG--EKAKDFAKAFGLKEPKVETWKFDWYED~LFAE
AN2493.1  	SPVNGSEAR-DGVPDTNFKFTVGKKG--QEGVPVTEFFDIEDAELENWQFSWYED1LFAQ
          	:* *****  :* ** .*..*: *..   :   .:. *  ::..:*.* *.*:** ***:

MG04900.1 	EAKTPSVVNVASKVHRRVALYEPGTYQVVLSYYGGKTTKATWVVRPLAEVKKTKNVIFFI
NCU01376.1	DAKTPSVVNVASKIYRQVALYEPGTYTVTLNYYSGKTTTATWVVRPLATKKKAKNVILFI
FG07375.1 	DQNKPSIVNVASKIYRKISLNEPGKYTVTLNYYNGEKTTAEWTVRPLQTKRKAKNVIFFI
AN2493.1  	DAETPSVVNVTSKAYRNLALYEPGEYEAILTYYGKEQTVANWLVRDIPKKRRAKNVVLFV
          	: :.**:***:** :*.::* *** * . *.**. : * * * ** :   :::***::*:

MG04900.1 	G~DGMT1TNM~ITAARLLGHKSINGKYQSTMQLDKFPVIGHQM~THSIDTYITDSANSAS
NCU01376.1	G1DGMT~TNM~ITAARLLAHKSINGKYQSTLQLDKFPTLGHQM0THSIDSFITDSANSAS
FG07375.1 	G~DGMT~TNM0ITAARLLGHKSINGKYQTLMKLDEFPVLGHQM~THSIDSYITDSANSAS
AN2493.1  	G~DGMT~TNM~ITAARLIAHKSINGKYQTKLQLDKFPVLGHQM~THSMDSFITDSANSAA
          	* **** *** ******:.*********: ::**:**.:**** ***:*::********:

MG04900.1 	ALYSGHKSTVNAMG~VYVDSSPDAFDDPKVETVVEMLKRVW~GSAWGAV2STAFIADATP
NCU01376.1	ALYTGHKTTVNAMG2VYVDSSPDKFDDPKVETIVELLKRIW0GSAWGAV~STAFIADATP
FG07375.1 	ALYSGHKSTVNAMG2VYADSSPDPFDDPKVETIVEIFKRIW0GGAWGAV~STAYLADATP
AN2493.1  	ALYGGHKTTVNCLG~VYADSSEDPFDDPKFELLPEIFRRQH~NGGIGIV~STAFLADATP
          	*** ***:***.:* **.*** * *****.* : *:::*   ... * * ***::*****

MG04900.1 	IALTAHTR2LRSQYGPLINQALNGVTNYTWTNHGGPDAYFGGGADQFIAGSASFEGKDYY
NCU01376.1	TALTAHTR~SRYEYGALIEQALNGFSNTSWTKQDGPDVYFGGGAEQFLPSSASYQGKDYY
FG07375.1 	IALTGHTR~LRGQYGPLIDQALNGNSNYSWTKHEGPDVFFGGGAENFIAGKGSYQGKDYY
AN2493.1  	GAMTSHTR~LRSEYDHVISTFLEGITNHTWTEFDGPDVLFGAGAENFLTSEDAP--RDYY
          	 *:*.***  * :*. :*.  *:* :* :**:  ***. **.**::*:... :   :***

MG04900.1 	DEFAKKGYTVSLNKTALEAIPNDQRALGIFCRGNLPVWLDRNVFTDYLGKQKNDPSGADK
NCU01376.1	AEFAKKGYSVSLNKTSLLALPDDKKALGIFCKSNLPVWLDRNIFPENTMKLKNDPSGASG
FG07375.1 	KEFQKKGYTVSHNKTSLLKADKSKRALGVFCRSNLPVWLDRNVFTDNIEGLENDPTGKEA
AN2493.1  	KLFTEKGYSLSLNKTSLESVSNEDRALGVFSLSNLPTWLDRNVYQDNLKELANHPDGSGR
          	  * :***::* ***:*    ...:***:*. .***.*****:: :      *.* *   

MG04900.1 	PALDLPGLKEMTLKAIDVLHTRGGEKGFFLMSEAASIDKQMHSLDYDRALGDLLELDDTV
NCU01376.1	PAKDIPGQKEMTLKAIDILHKRGGKDGFFLMSEAASIDKQMHVLDYERALGDLLELDDTV
FG07375.1 	DATDLPGLKDMTLKAIDVLSTRGKDKGFFLMSEAASIDKQMHALDYDRALGDLLELDDTV
AN2493.1  	DAEDLPGLKDMTVKAIDILDTRHSKEGWFLMAEAASIDKQMHSLDYDRSLGELLELDDTV
          	 * *:** *:**:****:* .*  ..*:***:********** ***:*:**:********

MG04900.1 	KATVEKLEKMGILNETLIVVSADHGHGFD~VFGSADTKYLDSKDN--DREKRNAVGVYER
NCU01376.1	RATIQKLQDLGELNNTLIVVTADHGHGFD~VFGGADTKYLAAQHDKDDRHKRSAVGVYHE
FG07375.1 	RATIEKLKKLDILDETLIIVSADHGHGFD2VWGSADTEYLSEQED--DRAKRRAIGTYEK
AN2493.1  	RATIERLDKLGILEDTLVLVTADHGHGFD~VTGSVDTVYLEQQED--DRDKRRAVGVYQN
          	:**:::*..:. *::**::*:******** * *..** **  :.:  ** ** *:*.*..

MG04900.1 	SGQSMYQVE-VPGVSYNTGPNFPVNWDPRYRIASGFGAMPDHREAYKLN-TRPRAPTTTT
NCU01376.1	SGLSQYTVE-QPGISYNTGPNFPVNWDPRYVIAAGTGAAPDRREQYGVGKNGSRTPAVTL
FG07375.1 	SGQSQYTKK-AKGINYGTGANFPTNWEPRYAIAGGVGAAPDRRENYKVH-KSPREAAVEL
AN2493.1  	SGLSQYTIGGSNALRYSEGVHFPARWDPRYAIHAGTVAFPDHRENYKVHKDGPRTPAEEG
          	** * *   .  .: *. * :**..*:*** * .*  * **:** * : .  .* .:   

MG04900.1 	PGRASNDMYANPADQPGGYMINGTLPPKEAQGVHSLTDVPVYAMGPCAETFGGTYNNVDI
NCU01376.1	PGMGDDDYYVNPTDRTGGFVVNGTLPTTESQGVHSLTDVPVFAQGPCSGNFGGVYSNTDI
FG07375.1 	K---DGDYYVNPEDAPDGFVINGTISTDNSQGVHSLTDVPVYALGPCQDTFGGTYSNIDI
AN2493.1  	G---DGTVYASYKDAVSGFLINGTLALDADQGVHSLTDVPVYARGPCQELFGGTINSIDI
          	    ..  *..  *  .*:::***:.    ***********:* ***   ***. .. **

MG04900.1 	FYKIASCLGLGHSKEKQNCASRKH-----
NCU01376.1	FFKIASCLGLGHPTKKTDGGKGR------
FG07375.1 	FYKIANCLGLAQGQKHGY-----------
AN2493.1  	FFNIAECLGLAETKRGKSNGGGGHRNSKQ
          	*::**.****..  .    .     .:..
```
